# Arginine, Transsulfuration, and Folic Acid Pathway Metabolomics in Chronic Obstructive Pulmonary Disease: A Systematic Review and Meta-Analysis

**DOI:** 10.3390/cells12172180

**Published:** 2023-08-30

**Authors:** Angelo Zinellu, Arduino A. Mangoni

**Affiliations:** 1 Department of Biomedical Sciences, University of Sassari, 07100 Sassari, Italy; azinellu@uniss.it; 2 Discipline of Clinical Pharmacology, College of Medicine and Public Health, Flinders University, Bedford Park, SA 5042, Australia; 3 Department of Clinical Pharmacology, Flinders Medical Centre, Southern Adelaide Local Health Network, Bedford Park, SA 5042, Australia

**Keywords:** folic acid, transsulfuration, oxidative stress, nitric oxide, biomarkers, chronic obstructive pulmonary disease, homocysteine, asymmetric dimethylarginine, symmetric dimethylarginine, ornithine

## Abstract

There is an increasing interest in biomarkers of nitric oxide dysregulation and oxidative stress to guide management and identify new therapeutic targets in patients with chronic obstructive pulmonary disease (COPD). We conducted a systematic review and meta-analysis of the association between circulating metabolites within the arginine (arginine, citrulline, ornithine, asymmetric, ADMA, and symmetric, SDMA dimethylarginine), transsulfuration (methionine, homocysteine, and cysteine) and folic acid (folic acid, vitamin B_6_, and vitamin B_12_) metabolic pathways and COPD. We searched electronic databases from inception to 30 June 2023 and assessed the risk of bias and the certainty of evidence. In 21 eligible studies, compared to healthy controls, patients with stable COPD had significantly lower methionine (standardized mean difference, SMD = −0.50, 95% CI −0.95 to −0.05, *p* = 0.029) and folic acid (SMD = −0.37, 95% CI −0.65 to −0.09, *p* = 0.009), and higher homocysteine (SMD = 0.78, 95% CI 0.48 to 1.07, *p* < 0.001) and cysteine concentrations (SMD = 0.34, 95% CI 0.02 to 0.66, *p* = 0.038). Additionally, COPD was associated with significantly higher ADMA (SMD = 1.27, 95% CI 0.08 to 2.46, *p* = 0.037), SDMA (SMD = 3.94, 95% CI 0.79 to 7.08, *p* = 0.014), and ornithine concentrations (SMD = 0.67, 95% CI 0.13 to 1.22, *p* = 0.015). In subgroup analysis, the SMD of homocysteine was significantly associated with the biological matrix assessed and the forced expiratory volume in the first second to forced vital capacity ratio, but not with age, study location, or analytical method used. Our study suggests that the presence of significant alterations in metabolites within the arginine, transsulfuration, and folic acid pathways can be useful for assessing nitric oxide dysregulation and oxidative stress and identifying novel treatment targets in COPD. (PROSPERO registration number: CRD42023448036.)

## 1. Introduction

The global public health and financial burden of chronic obstructive pulmonary disease (COPD) remains unacceptably high despite the availability of different pharmacological and non-pharmacological treatments in this ever-increasing patient group [1,2,3,4,5,6,7]. Such challenges have stimulated a significant body of research to better understand the molecular, biochemical, and cellular mechanisms underpinning the pathophysiology of COPD and identify novel druggable targets and therapies [8,9,10,11]. Whilst the role of local (airway) and systemic inflammation in COPD is well established, using conventional biomarkers (e.g., C-reactive protein) and specific blood cell types [12,13,14,15], studies have also focused on the dysregulation of the endogenous messenger nitric oxide (NO) and the redox state [16,17,18,19,20,21,22,23,24]. The investigation of possible alterations in the NO pathway and redox balance are also important in this context given their involvement in other disease states, some of them, e.g., atherosclerosis and cardiovascular disease, frequently associated with COPD [25,26,27,28,29,30,31]. For example, in epidemiological studies, the prevalence of atherosclerotic cardiovascular disease in patients with COPD has been shown to range between 20% and 60% [32,33,34]. Furthermore, the coexistence of COPD and cardiovascular disease is associated with poorer quality of life and functional capacity and a higher risk of COPD exacerbations, hospitalizations, and mortality [35,36,37,38].

**Figure 1:**
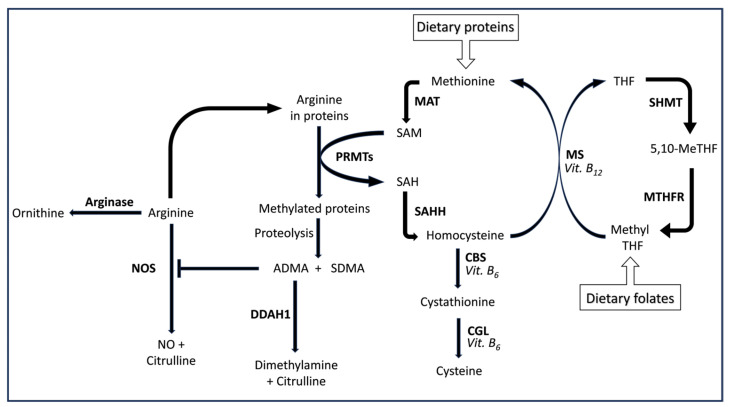
Schematic representation of the arginine, transsulfuration, and folic acid metabolic pathways. 5,10-MeTHF, 5,10-methylenetetrahydrofolate; CBS, cystathionine β-synthase; CGL, cystathionine γ-lyase; ADMA, asymmetric dimethylarginine; SDMA, symmetric dimethylarginine; DDAH1, dimethylarginine dimethylaminohydrolase 1; MAT, methionine adenosyltransferase; MHTFR, 5,10-methylenetetrahydrofolate reductase; MS, methionine synthase; NOS, nitric oxide synthase; PRMTs, protein arginine methyltransferases; SAH, S-adenosyl-homocysteine; SAM, S-adenosyl-methionine; SAHH, S-Adenosylhomocysteine hydrolase; SHMT, serine hydroxymethyltransferase. CBS and CGL are vitamin B_6_ dependent; methionine synthase is vitamin B_12_ dependent.

A significant limitation in the development of analytical platforms for the assessment of NO and biomarkers of oxidative stress in biological samples is represented by the highly reactive nature of these compounds, the relatively short half-life of NO, and the influence of other factors in the assessment of circulating NO metabolites such as nitrite and nitrate [39,40,41,42,43,44,45]. Therefore, an alternative approach consists of measuring stable metabolites within metabolic pathways that are closely associated with NO synthesis and oxidative stress. In this context, several metabolites within the arginine, transsulfuration, and folic acid metabolic pathways have been shown to reflect alterations in NO synthesis and/or redox state. Furthermore, these metabolites can be measured in serum or plasma using a wide range of analytical methods for targeted metabolomic analysis, involving the assessment of pre-defined metabolites within specific biochemical pathways (Figure 1) [46,47,48,49,50,51,52,53,54]. The arginine pathway includes (a) arginine, a critical amino acid and substrate for several enzymes, e.g., protein arginine methyltransferases (PRMTs), arginase 1 and 2, and NO synthases (NOS) [46,55]; (b) citrulline, the end product of enzymatic reactions catalyzed by NOS and isoform 1 of dimethylarginine dimethylaminohydrolase (DDAH1) [46,56]; (c) the methylated arginine analogues, asymmetric (ADMA) and symmetric (SDMA) methylarginine, which directly (ADMA) or indirectly (SDMA) downregulate NO synthesis [55,56,57,58,59]; and (d) ornithine, the end product of arginase 1 and 2 (Figure 1) [46,60]. The transsulfuration pathway regulates sulfur metabolism and redox balance and primarily involves the transfer of sulfur from homocysteine, a highly reactive amino acid derived from the dietary compound, methionine, to cysteine through the intermediate cystathionine, in enzymatic reactions that require vitamin B_6_ (Figure 1) [48,61]. Finally, the folic acid pathway plays a critical role in regulating several intracellular homeostatic mechanisms that also include the lowering of homocysteine concentrations through the regeneration of methionine in enzymatic reactions that involve vitamin B_12_ (Figure 1) [62,63].

Importantly, the known associations between the arginine, transsulfuration, and folic acid pathways, vascular homeostasis, and cardiovascular outcomes might also allow investigating the complex interplay between COPD, NO, oxidative stress, and atherosclerotic cardiovascular disease [55,56,58,64,65,66,67,68,69,70,71,72,73,74]. This knowledge would be potentially useful for identifying new therapeutic targets and management approaches in patients with COPD.

We investigated this issue by (a) appraising the available evidence, through a systematic review and meta-analysis, of the association between the circulating concentrations of key metabolites within the arginine, transsulfuration, and folic acid metabolic pathways and COPD, and (b) assessing, where possible, the relationship between the effect size of the observed differences vs. healthy controls and clinical and demographic characteristics.

## 2. Materials and Methods

### 2.1. Study Selection

A systematic search of publications was conducted in the electronic databases PubMed, Web of Science, and Scopus from inception to 30 June 2023. The search utilized the following terms and their combinations: “COPD” OR “chronic obstructive pulmonary disease” AND “methionine” OR “homocysteine” OR “cysteine” OR “cystathionine” OR “S-adenosylmethyonine” OR “S-adenosylhomocysteine” OR “S-adenosyl-methyonine” OR “S-adenosyl-homocysteine” OR “betaine” OR “dimethylglycine” OR “folates” OR “folic acid” OR “B_12_” OR “cobalamin” OR “B_6_” OR “pyridoxine” OR “arginine” OR “asymmetric dimethylarginine” OR “ADMA” OR “symmetric dimethylarginine” OR “citrulline” OR “ornithine”.

Two investigators independently screened the abstracts, full-text articles, and relevant references according to the following inclusion criteria: (a) the assessment of homocysteine, cysteine, methionine, vitamin B_6_, vitamin B_12_, folic acid, arginine, ADMA, SDMA, citrulline, or ornithine in plasma or serum, (b) the study of patients with stable COPD and healthy controls using a case–control design, (c) the inclusion of participants ≥18 years, and (d) the availability of full text in English language. The main exclusion criterion was the assessment of patients with acute exacerbations of COPD. The two investigators independently extracted the following variables into an electronic spreadsheet for further analysis: year of publication, first author, study country, participant number, age, male to female ratio, forced expiratory volume in the first second (FEV_1_), FEV_1_/forced vital capacity (FVC), biological matrix (plasma or serum), and analytical method used. A third investigator was involved in case of disagreement.

The Joanna Briggs Institute Critical Appraisal Checklist was used to assess the risk of bias [75], whereas the Grades of Recommendation, Assessment, Development, and Evaluation (GRADE) Working Group system was used to assess the certainty of evidence [76]. The Preferred Reporting Items for Systematic Reviews and Meta-Analyses 2020 statement was followed to present the results [77], and the International Prospective Register of Systematic Reviews was used to register our review (PROSPERO registration number: CRD42023448036).

### 2.2. Statistical Analysis

We created forest plots of standardized mean differences (SMDs) and 95% confidence intervals (CIs) (*p*-value < 0.05 for statistical significance), and estimated means and standard deviations from medians and interquartile ranges or ranges [78,79], or using the Graph Data Extractor software beta version (San Diego, CA, USA). The heterogeneity of SMD was evaluated using the Q statistic (significance level set at *p* < 0.10) [80,81]. Sensitivity analysis was used to assess the stability of the results [82]. The Egger’s and Begg’s tests and the “trim-and-fill” method were used to assess publication bias [83,84,85]. Univariate meta-regression and subgroup analyses investigated associations between the effect size and the following parameters: year of publication, study continent, sample size, age, male to female ratio, FEV_1_, FEV_1_/FVC, biological matrix, and analytical method used. Statistical analyses were performed using Stata 14 (Stata Corp., College Station, TX, USA).

## 3. Results

### 3.1. Literature Search

**Figure 2:**
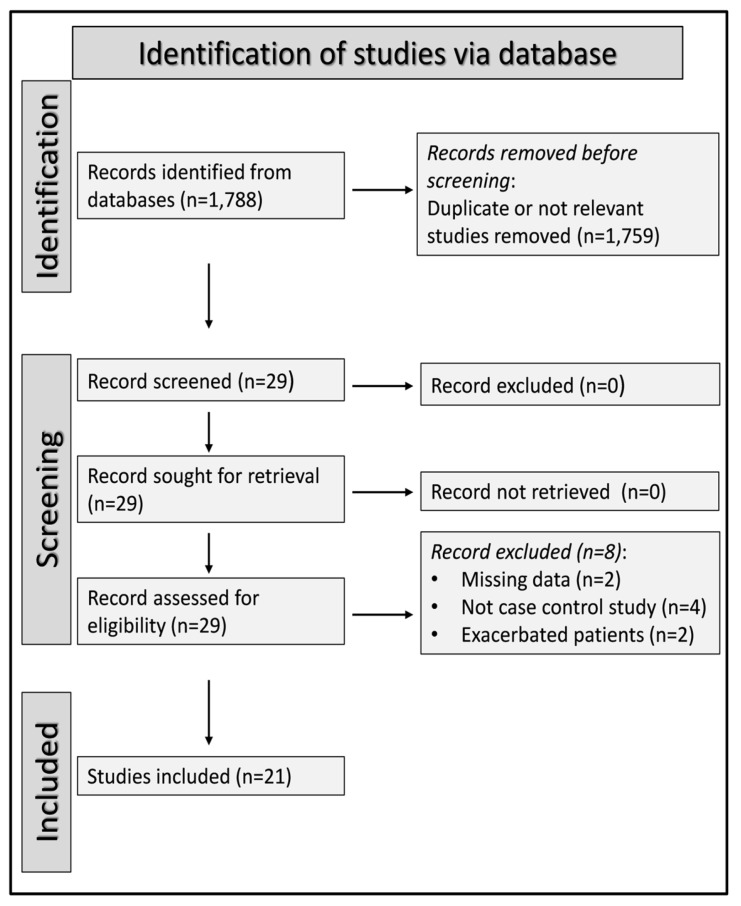
PRISMA 2020 flow diagram.

From a total of 1788 articles, we excluded 1759, as they were either duplicates or irrelevant. A full-text revision of the remaining 29 articles led to the exclusion of further eight because they had missing data (n = 2), unsuitable (not case–control) design (n = 4), or included patients with acute exacerbation of COPD (n = 2). The 21 studies included in the final analysis were published between 1998 and 2020 (Figure 2 and Table 1) [86,87,88,89,90,91,92,93,94,95,96,97,98,99,100,101,102,103,104,105,106]. There was no disagreement between the two independent investigators; therefore, input from a third investigator was not required. The cross-sectional design of all studies was primarily responsible for the initial low level of certainty given (rating 2, ⊕⊕⊝⊝). The risk of bias was low in all studies (Supplementary Table S3) [86,87,88,89,90,91,92,93,94,95,96,97,98,99,100,101,102,103,104,105,106].

### 3.2. Homocysteine

Homocysteine was measured in 11 studies investigating a total of 610 COPD patients (mean age: 57 years, 72% males) and 468 healthy controls (mean age: 44 years, 66% males) [87,89,90,91,92,93,99,100,104,105,106], six conducted in Asia [89,93,99,100,104,105], four in Europe [87,90,92,106], and one in Africa [91]. Liquid chromatography was used in four studies [87,89,91,92], an enzyme-linked immunosorbent assay in two [93,104], capillary electrophoresis laser induced with fluorescence detection in one [106], and a fluorescence polarization immunoassay in the remaining one [90]. No information regarding the analytical method was reported in three studies [99,100,105]. In liquid chromatography studies, two used a fluorimetric detector [89,92], and the remaining two used an ultraviolet detector [87,91]. Homocysteine was measured in plasma in eight studies [87,89,90,92,93,104,105,106], and in serum in the remaining three [91,99,100]. The FEV_1_ was reported in eight studies (range between 39% and 70%) [89,90,92,99,100,104,105,106], and the FEV_1_/FVC in five (range between 53% and 68%) [90,92,99,105,106].

**Figure 3:**
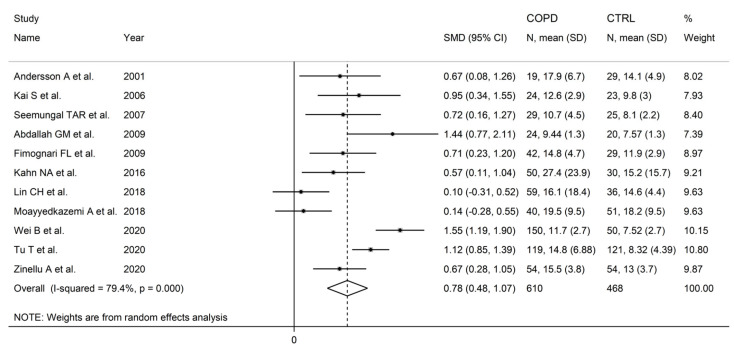
Forest plot of homocysteine concentrations in COPD patients and controls [87,89,90,91,92,93,99,100,104,105,106].

**Figure 4:**
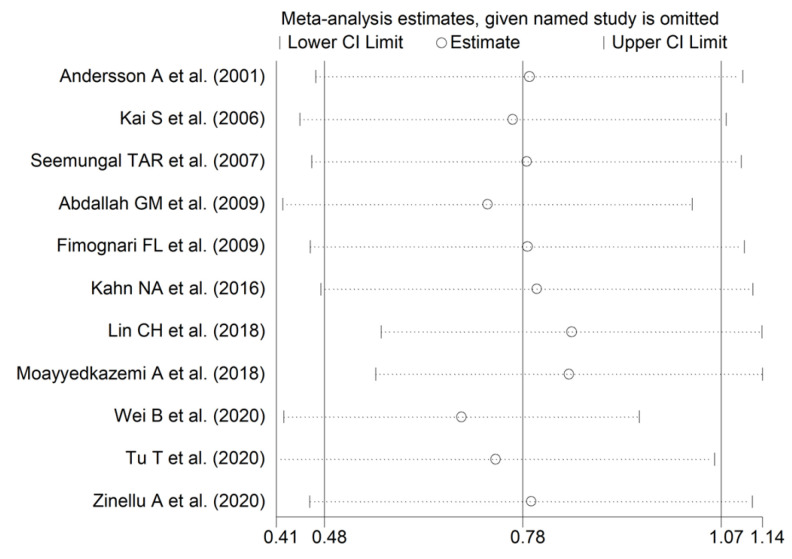
Sensitivity analysis of the association between homocysteine and COPD [87,89,90,91,92,93,99,100,104,105,106].

**Figure 5:**
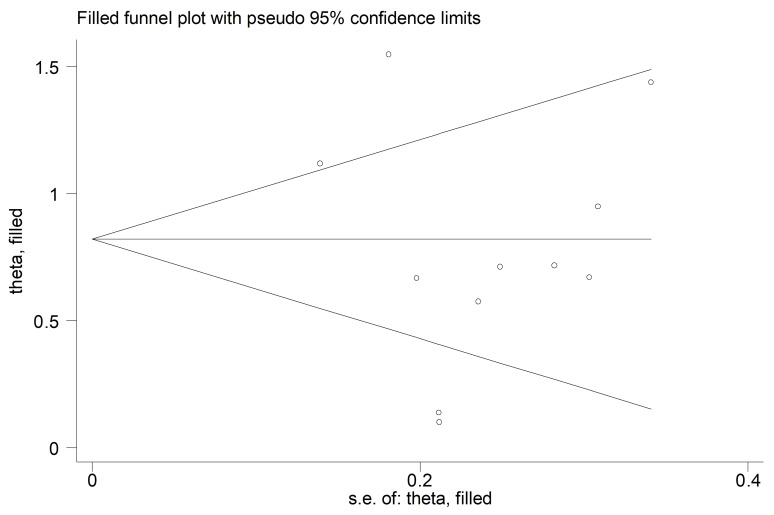
Funnel plot of studies investigating homocysteine in COPD after “trimming-and-filling”.

Homocysteine concentrations were significantly higher in COPD patients compared to controls (SMD = 0.78, 95% CI 0.48 to 1.07, *p* < 0.001; I^2^ = 79.4%, *p* < 0.001; Figure 3). The results were stable in sensitivity analysis (SMD range between 0.69 and 0.85; Figure 4). There was no publication bias (Begg’s test, *p* = 0.64); Egger’s test, *p* = 0.51). No additional study was identified using the “trim-and-fill” method (Figure 5).

**Figure 6:**
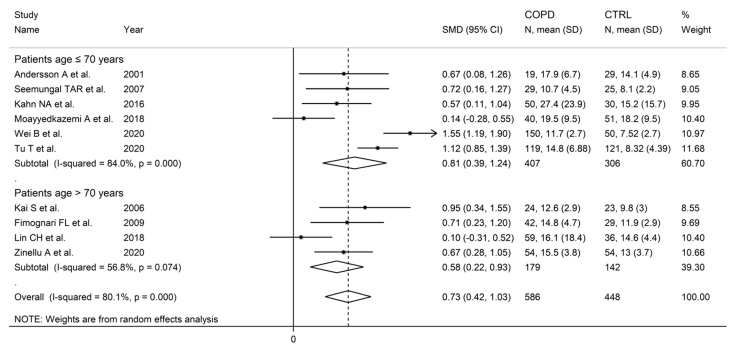
Forest plot of studies investigating homocysteine concentrations in COPD patients and controls according to patient age (≤70 years or ˃70 years) [87,89,90,92,93,99,100,104,105,106].

**Figure 7:**
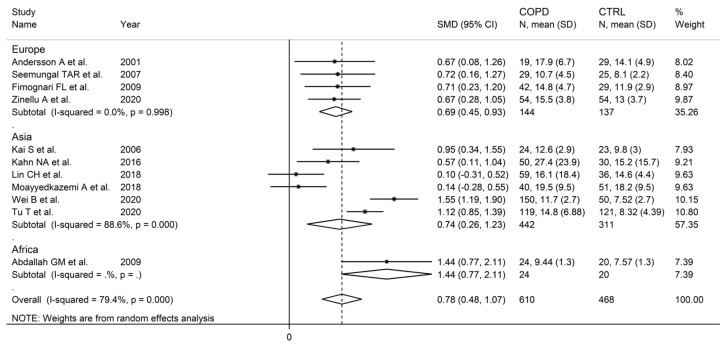
Forest plot of studies investigating homocysteine concentrations in COPD patients and controls according to study continent [87,89,90,91,92,93,99,100,104,105,106].

**Figure 8:**
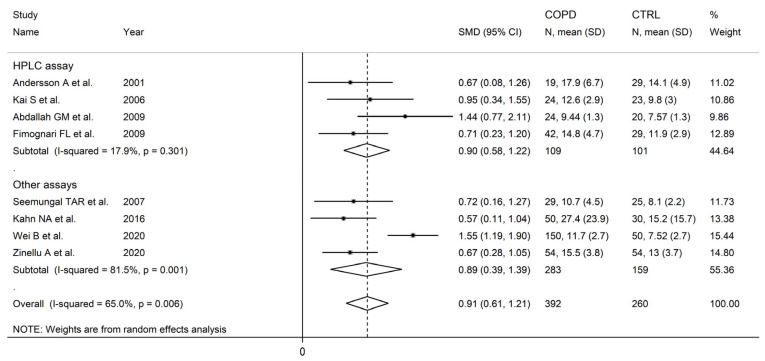
Forest plot of studies investigating homocysteine concentrations in COPD patients and controls according to analytical method [87,89,90,91,92,93,104,106].

**Figure 9:**
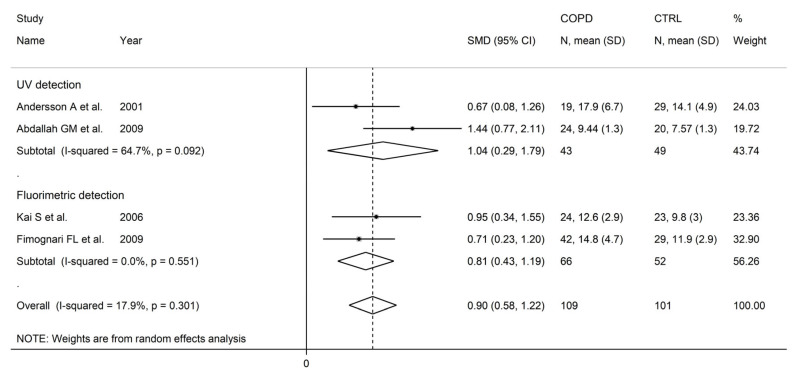
Forest plot of studies investigating homocysteine concentrations in COPD patients and controls according to the detection method used with liquid chromatography [87,89,91,92].

**Figure 10:**
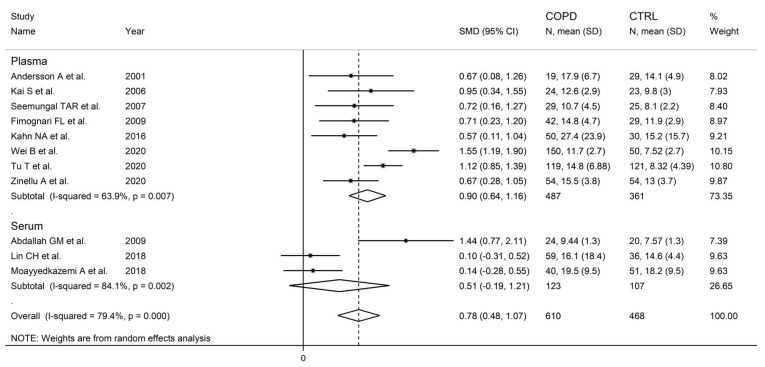
Forest plot of studies investigating homocysteine concentrations in COPD patients and controls according to measurement in serum or plasma [87,89,90,91,92,93,99,100,104,105,106].

**Figure 11:**
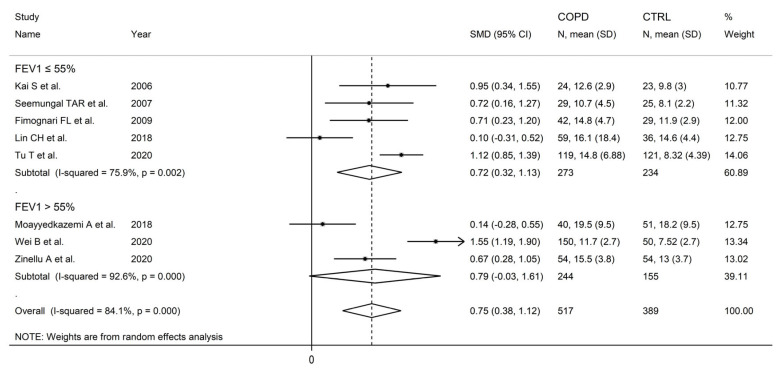
Forest plot of studies investigating homocysteine concentrations in COPD patients and controls according to FEV_1_ (≤55% or ˃55% years) [89,90,92,99,100,104,105,106].

**Figure 12:**
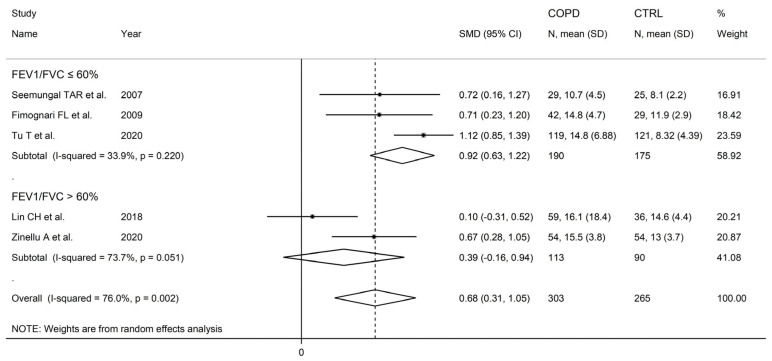
Forest plot of studies examining homocysteine concentration in COPD patients and controls according to FEV_1_/FVC (≤60% vs. ˃60%) [90,92,99,105,106].

There were no significant associations in meta-regression between the effect size and male to female ratio (t = −0.36, *p* = 0.73), number of participants (t = 1.39, *p* = 0.20), or publication year (t = 0.10, *p* = 0.92). In subgroup analysis, no significant differences (*p* = 0.47) in the pooled SMD were observed between studies in patients ≤70 years (SMD = 0.81, 95% CI 0.39 to 1.24, *p* < 0.001; I^2^ = 84.0%, *p* < 0.001), or >70 years (SMD = 0.58, 95% CI 0.22 to 0.93, *p* = 0.001; I^2^ = 56.8%, *p* = 0.074; Figure 6), with a lower between-study variance in the >70 years subgroup. Similarly, no significant differences (*p* = 0.86) in effect size were observed between studies conducted in Europe (SMD = 0.69, 95% CI 0. 45 to 0.93, *p* < 0.001; I^2^ = 0.0%, *p* = 0.998) and Asia (SMD = 0.74, 95% CI 0.26 to 1.23, *p* = 0.003; I^2^ = 88.6%, *p* < 0.001; Figure 7), with a virtually absent heterogeneity in the European subgroup. Additionally, no significant differences (*p* = 0.95) in the pooled SMD were observed between studies using high performance liquid chromatography (SMD = 0.90, 95% CI 0.58 to 1.22, *p* < 0.001; I^2^ = 17.9%, *p* = 0.30) and other methods (SMD = 0.89, 95% CI 0.39 to 1.39, *p* < 0.001; I^2^ = 81.5%, *p* = 0.001; Figure 8), with a lower between-study variance in the liquid chromatography subgroup. Among the liquid chromatography studies, no significant differences (*p* = 0.64) in the pooled SMD were observed between studies using ultraviolet detection (SMD = 1.04, 95% CI 0.29 to 1.79, *p* = 0.007; I^2^ = 64.7%, *p* = 0.092), and fluorimetric detection (SMD = 0.81 95% CI 0.43 to 1.19, *p* = 0.001; I^2^ = 0.0%, *p* = 0.55; Figure 9), with a virtually absent heterogeneity in the fluorimetric detection subgroup. The pooled SMD was statistically significant in studies assessing plasma (SMD = 0.90, 95% CI 0.64 to 1.16, *p* < 0.001; I^2^ = 63.9%, *p* = 0.007), but not serum (SMD = 0.51, 95% CI −0.19 to 1.21, *p* = 0.16; I^2^ = 84.1%, *p* = 0.002; Figure 10). Furthermore, the pooled SMD was statistically significant in studies of patients with FEV_1_ ≤55% (SMD = 0.72, 95% CI 0.32 to 1.13, *p* < 0.001; I^2^ = 75.9%, *p* = 0.002), but not FEV_1_ ˃55% (SMD = 0.79, 95% CI −0.03 to 1.61, *p* = 0.06; I^2^ = 92.6%, *p* < 0.001; Figure 11). Finally, the pooled SMD was statistically significantly in studies of patients with FEV_1_/FVC ≤60% (SMD = 0.92, 95% CI 0.63 to 1.22, *p* < 0.001; I^2^ = 33.9%, *p* = 0.22), but not FEV1/FVC ˃60% (SMD = 0.39, 95% CI −0.16 to 0.94, *p* = 0.17; I^2^ = 73.7%, *p* = 0.051; Figure 12), with a lower heterogeneity in the FEV_1_/FVC ≤60% subgroup.

The level of certainty remained low (rating 2, ⊕⊕⊝⊝) after considering the low risk of bias in all studies, the high but partially explainable heterogeneity, the lack of indirectness, the relatively low imprecision, the moderate effect size, and the lack of publication bias.

### 3.3. Cysteine

Cysteine was measured plasma in two European studies including a total of 73 COPD patients (mean age: 72 years, 66% males) and 83 healthy controls (mean age: 70 years, 65% males) [87,106]. Liquid chromatography with ultraviolet detection was used in one study [87], and capillary electrophoresis with laser-induced fluorescence in the other [106].

**Figure 13:**
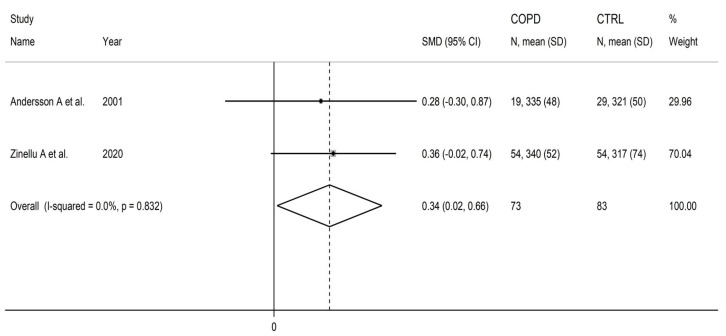
Forest plot of studies investigating cysteine concentrations in COPD patients and controls [87,106].

Cysteine concentrations were significantly higher in COPD patients compared to controls (SMD = 0.34, 95% CI 0.02 to 0.66, *p* = 0.038; I^2^ = 0.0%, *p* = 0.83; Figure 13). The limited number of studies prevented sensitivity analysis, the assessment of publication bias, and the conduct of meta-regression and subgroup analyses.

The level of certainty was downgraded to very low (rating 1, ⊕⊝⊝⊝) after considering the low risk of bias in all studies, the virtually absent heterogeneity, the lack of indirectness, the relatively low imprecision, the relatively small effect size, and the lack of assessment of publication bias (downgrade one level).

### 3.4. Methionine

Two studies investigated plasma methionine in a total of 42 COPD patients and 38 healthy controls [86,88]. One study was conducted in Europe [86], and the other in Asia one [88]. Liquid chromatography with fluorimetric detection was used in both studies [86,88].

**Figure 14:**
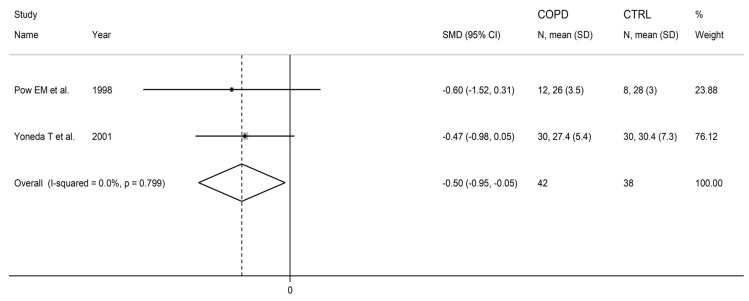
Forest plot of studies investigating methionine concentrations in COPD patients and controls [86,88].

Methionine concentrations were significantly lower in COPD patients compared to controls (SMD = −0.50, 95% CI −0.95 to −0.05, *p* = 0.029; I^2^ = 0.0%, *p* = 0.80; Figure 14). The limited number of studies prevented sensitivity analysis, the assessment of publication bias, and the conduct of meta-regression and subgroup analyses.

The level of certainty was downgraded to very low (rating 1, ⊕⊝⊝⊝) after considering the low risk of bias in all studies, the virtually absent heterogeneity, the lack of indirectness, the relatively low imprecision, the relatively moderate effect size, and the lack of assessment of publication bias (downgrade one level).

### 3.5. Vitamin B_12_

Vitamin B_12_ was measured in three studies including a total of 125 patients (mean age: 71 years, 84% males) and 85 healthy controls (mean age: 71 years, 83% males) [91,92,99]. One study was conducted in Africa [91], one in Europe [92], and one in Asia [99]. One study used liquid chromatography with ultraviolet detection [91], the second a chemiluminometric immunoassay [92], and the third did not provide relevant details regarding the analytical method used [99]. Two studies assessed serum [91,92], and the third plasma [99].

**Figure 15:**
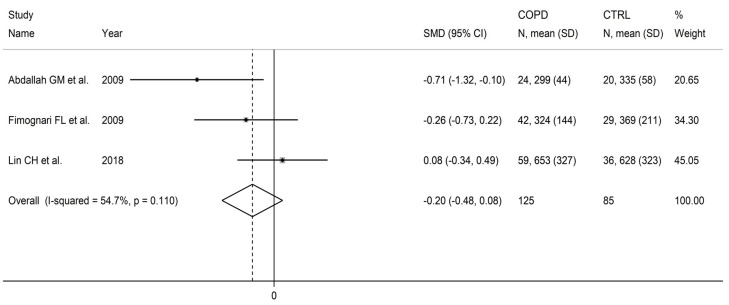
Forest plot of studies investigating vitamin B_12_ concentrations in COPD patients and controls [91,92,99].

There were non-significant differences in vitamin B_12_ concentrations between COPD patients and controls (SMD = −0.20, 95% CI −0.48 to 0.08, *p* = 0.16; I^2^ = 54.7%, *p* = 0.11; Figure 15). The limited number of studies prevented sensitivity analysis, the assessment of publication bias, and the conduct of meta-regression and subgroup analyses.

The level of certainty was downgraded to very low (rating 1, ⊕⊝⊝⊝) after considering the low risk of bias in all studies, the moderate heterogeneity, the lack of indirectness, and the lack of assessment of publication bias (downgrade one level).

### 3.6. Folic Acid

Three studies measured folic acid in a total of 125 COPD patients (mean age: 71 years, 84% males) and 85 healthy controls (mean age: 71 years, 83% males) [91,92,99]. One study was conducted in Africa [91], one in Europe [92], and one in Asia [99]. One study used liquid chromatography with ultraviolet detection [91], the second a chemiluminometric immunoassay [92], and the third did not provide relevant details regarding the analytical method used [99]. Two studies assessed serum [91,92], and the third assessed plasma [99].

**Figure 16:**
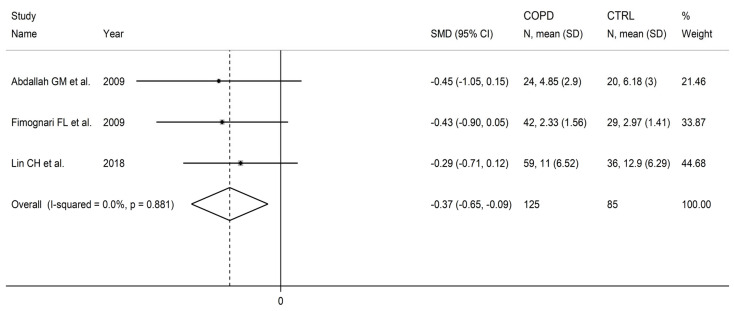
Forest plot of studies investigating folic acid concentrations in COPD patients and controls [91,92,99].

Folic acid concentrations were significantly lower in COPD patients compared to controls (SMD = −0.37, 95% CI −0.65 to −0.09, *p* = 0.009; I^2^ = 0.0%, *p* = 0.88; Figure 16). The limited number of studies prevented sensitivity analysis, the assessment of publication bias, and the conduct of meta-regression and subgroup analyses.

The level of certainty was downgraded to very low (rating 1, ⊕⊝⊝⊝) after considering the low risk of bias in all studies, the virtually absent heterogeneity, the lack of indirectness, the relatively low imprecision, the relatively moderate effect size, and the lack of assessment of publication bias (downgrade one level).

### 3.7. Arginine

Arginine was measured in four studies including a total of 117 COPD patients (mean age: 67 years) and 111 healthy controls (mean age: 64 years) [86,88,94,95]. Three were conducted in Europe [86,94,95], and the remaining one in Asia [88]. Three studies used liquid chromatography with fluorimetric detection [86,88,94], and the remaining one capillary electrophoresis with ultraviolet detection [23]. Three studied assessed plasma [88,94,95], whilst the remaining one assessed serum [86].

**Figure 17:**
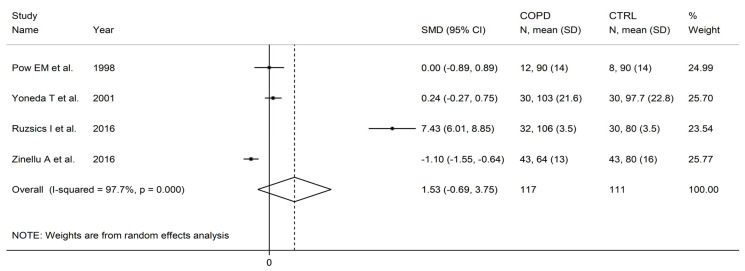
Forest plot of studies investigating arginine concentrations in COPD patients and controls [86,88,94,95].

There were non-significant between-group differences in arginine concentrations (SMD = 1.53, 95% CI −0.69 to 3.75, *p* = 0.18; I^2^ = 97.7%, *p* < 0.001; Figure 17). The limited number of studies prevented sensitivity analysis, the assessment of publication bias, and the conduct of meta-regression and subgroup analyses.

The level of certainty was downgraded to extremely low (rating 0, ⊝⊝⊝⊝) after considering the low risk of bias in all studies, the high and unexplained heterogeneity (downgrade one level), the lack of indirectness), and the lack of assessment of publication bias (downgrade one level).

### 3.8. Asymmetric Dimethylarginine

ADMA was measured in six studies including a total of 314 COPD patients (mean age: 67 years, males 69%) and 218 healthy controls (mean age: 66 years, males 63%) were evaluated [94,95,96,97,98,101]. Four studies were conducted in Europe [94,95,97,98], and two in Asia [96,101]. Four studies used liquid chromatography [94,97,98,101], one capillary electrophoresis with ultraviolet detection [95], and the remaining one used an enzyme-linked immunosorbent assay [96]. Among the liquid chromatography studies, three utilized a fluorimetric detection [94,98,101], whereas the remaining one did not provide relevant information [97]. Plasma was assessed in four studies [94,96,97,101], and serum in the remaining two [95,98].

**Figure 18:**
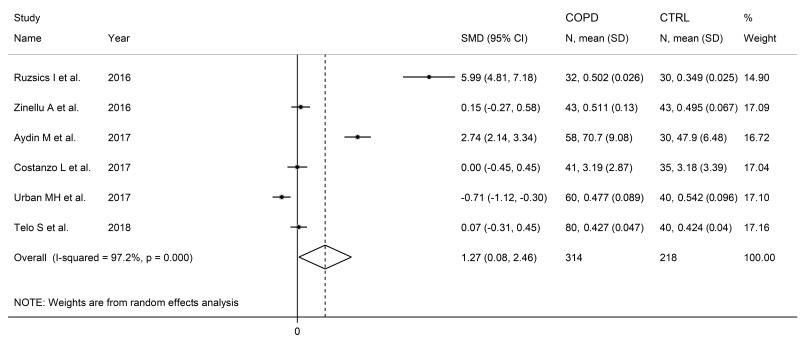
Forest plot of studies investigating ADMA concentrations in COPD patients and controls [94,95,96,97,98,101].

ADMA concentrations were significantly higher in COPD patients compared to controls (SMD = 1.27, 95% CI 0.08 to 2.46, *p* = 0.037; I^2^ = 97.2%, *p* < 0.001; Figure 18). The limited number of studies prevented sensitivity analysis, the assessment of publication bias, and the conduct of meta-regression and subgroup analyses.

The level of certainty was downgraded to very low (rating 1, ⊕⊝⊝⊝) after considering the low risk of bias in all studies, the high and unexplained heterogeneity (downgrade one level), the lack of indirectness, the relatively low imprecision, the relatively large effect size (upgrade one level), and the lack of assessment of publication bias (downgrade one level).

### 3.9. Symmetric Dimethylarginine

Three European studies measured SDMA in a total of 104 COPD patients (mean age: 67 years, males 59%) and 88 healthy controls (mean age: 62 years, males 63%) [94,95,102]. Two studies used liquid chromatography with fluorimetric detection [94,102], and the remaining one used capillary electrophoresis with ultraviolet detection [95]. Two studies assessed serum [94,102], and the remaining one used plasma [95].

**Figure 19:**
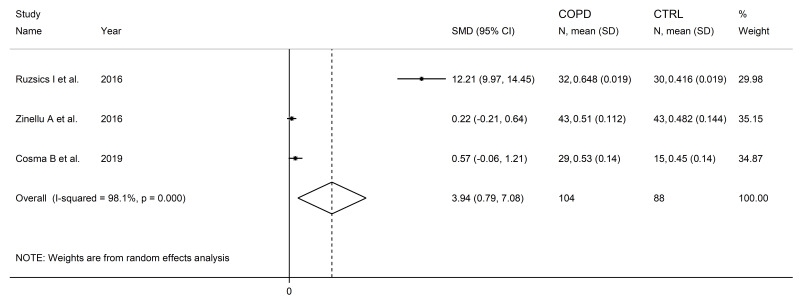
Forest plot of studies investigating SDMA concentrations in COPD patients and controls [94,95,102].

SDMA concentrations were significantly higher in COPD patients compared to controls (SMD = 3.94, 95% CI 0.79 to 7.08, *p* = 0.014; I^2^ = 98.1%, *p* < 0.001; Figure 19). The limited number of studies prevented sensitivity analysis, the assessment of publication bias, and the conduct of meta-regression and subgroup analyses.

The level of certainty was downgraded to very low (rating 1, ⊕⊝⊝⊝) after considering the low risk of bias in all studies, the high and unexplained heterogeneity (downgrade one level), the lack of indirectness, the relatively low imprecision, the relatively large effect size (upgrade one level), and the lack of assessment of publication bias (downgrade one level).

### 3.10. Ornithine

Plasma ornithine was measured in three studies including a total of 117 COPD patients (mean age: 69 years) and 82 healthy controls (mean age: 58 years) [86,88,103]. Two studies were conducted in Asia [88,103], and one in Europe [86]. Liquid chromatography with fluorimetric detection was used in two studies [86,88], and liquid chromatography with ultraviolet detection was used in the remaining one [103].

**Figure 20:**
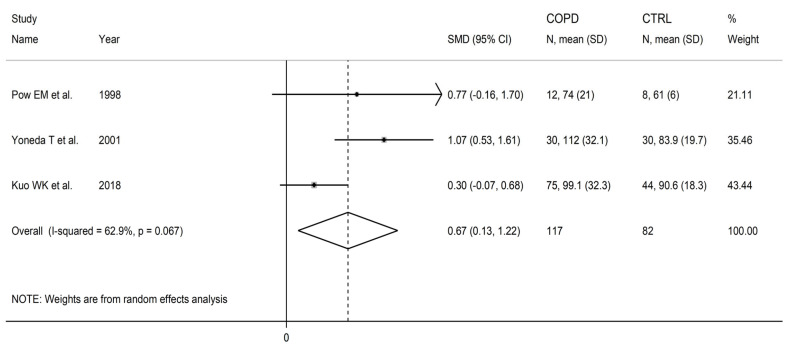
Forest plot of studies investigating ornithine concentrations in COPD patients and controls [86,88,103].

Ornithine concentrations were significantly higher in COPD patients than controls (SMD = 0.67, 95% CI 0.13 to 1.22, *p* = 0.015; I^2^ = 62.9%, *p* = 0.067; Figure 20). The limited number of studies prevented sensitivity analysis, the assessment of publication bias, and the conduct of meta-regression and subgroup analyses.

The level of certainty was downgraded to very low (rating 1, ⊕⊝⊝⊝) after considering the low risk of bias in all studies, the moderate heterogeneity, the lack of indirectness, the relatively low imprecision, the moderate effect size, and the lack of assessment of publication bias (downgrade one level).

### 3.11. Vitamin B_6_ and Citrulline

In a study comparing 42 COPD patients (71 ± 8 years) and 29 age-matched healthy controls (71 ± 6 years), COPD patients had significantly lower vitamin B_6_ concentrations compared to controls (5.6 ± 5.1 vs. 9.1 ± 6.4 pg/mL, *p* = 0.036) using a radioimmunoassay method [92].

In a study comparing 12 COPD patients (66 ± 2 years) and eight age-matched healthy controls (64 ± 3 years), there were non-significant differences in plasma citrulline concentrations between the two groups (48 ± 6 vs. 54 ± 7 μmol/L) using a liquid chromatography assay with fluorimetric detection [86].

## 4. Discussion

We observed significant alterations in the circulating concentrations of key metabolites within the arginine, transsulfuration, and folic acid metabolic pathways in COPD using targeted metabolomic analysis. Compared to healthy controls, patients with stable COPD had significantly lower concentrations of methionine and folic acid, and higher concentrations of homocysteine and cysteine. In the context of arginine pathways, COPD was also associated with significant elevations of ADMA, SDMA, and ornithine. Subgroup analysis, which was only possible for studies investigating homocysteine, showed that the SMD of this metabolite was significantly associated with the biological matrix assessed (plasma vs. serum) and the FEV_1_ to FVC ratio, but not with age, study location, or analytical method used.

Homocysteine, a highly reactive sulfur-containing amino acid and a metabolite of methionine (Figure 1), has been extensively investigated in view of its capacity to disrupt vascular homeostasis through the inhibition of NO synthesis, endothelial dysfunction, and stimulation of pro-inflammatory and pro-oxidative pathways in the vascular wall and systemically [68,107,108,109,110,111,112,113,114,115]. Not surprisingly, higher circulating homocysteine concentrations have been associated with an increased risk of cardiovascular morbidity and mortality in several observational studies [68,116,117]. Notably, homocysteine can also inhibit DDAH1 with a consequent accumulation of ADMA [109], whereas folic acid and vitamin B_12_ stimulate the conversion of homocysteine into methionine [68], with consequent homocysteine lowering. These effects further highlight the complex interplay between the arginine, transsulfuration, and folic acid metabolic pathways (Table 1).

The results of our systematic review and meta-analysis, particularly the increased circulating concentrations of homocysteine and ADMA, and the reduced concentrations of folic acid and methionine suggests a significant dysregulation of these pathways in COPD. Such dysregulation would manifest biologically as an impaired synthesis of NO via ADMA accumulation, a pro-oxidative state via homocysteine accumulation, and an overall pro-atherosclerotic state. Furthermore, epidemiological studies have reported that higher ADMA concentrations are independently associated with a significant reduction in FEV_1_ and FVC [118]. Similar negative associations with FEV_1_ and FVC have been reported specifically in healthy smokers [119]. In further support of these observations, a study has also reported that patients with COPD have a significantly lower dietary intake of folic acid compared to healthy controls (231 ± 90 vs. 261 ± 110 μg/day, *p* < 0.001) [120]. Notably, in this study, COPD patients in the upper quartile of folic acid intake had significantly lower breathlessness and higher FEV_1_ and FVC values compared to patients in the bottom quartile. In a more recent nationwide survey of COPD patients, folic acid concentrations were positively associated with FEV_1_ and FVC values, particularly in males and in current smokers [121]. Given the well-known homocysteine-lowering effects of folic acid supplementation [68,71,112], and the emerging evidence of additional lowering effects on circulating ADMA [122,123,124,125,126], further studies are warranted to determine whether folic acid supplementation, with or without vitamin B_12_, can improve symptoms, lung function, and clinical outcomes in patients with COPD.

The observed increases in circulating SDMA and cysteine in COPD are intriguing. Like ADMA, SDMA is derived from the methylation of arginine residues in proteins by PRMT 2 [127,128] (Figure 1). However, unlike ADMA, SDMA does not directly inhibit NOS nor is metabolized by DDAH1 and is eliminated in the urine unchanged [56,58]. In experimental studies, SDMA has been shown to indirectly reduce NO availability by favoring the uncoupling of NOS and by competing with the transport of the essential NOS substrate arginine [129,130,131]. The relatively high prevalence of chronic kidney disease in patients with COPD might potentially account for the reduced renal elimination and consequent accumulation of SDMA in this group [132,133,134]. However, recent studies have also reported an association between COPD and PRMTs. For example, an increased expression of PRMT 7, which has also been demonstrated to synthesize SDMA [135,136,137], has been observed in lung tissue macrophages of patients with COPD. Furthermore, a reduced expression of PRMT 7 in mice models of COPD was associated with a reduction in markers of lung injury [137]. The increase in cysteine concentrations in COPD is counterintuitive, given that this thiol is essential for protein synthesis, exerts antioxidant effects, and is a precursor of the major antioxidant glutathione and another metabolite with antioxidant effects, taurine [48,138,139,140]. Additional research is required to confirm these findings and elucidate the mechanisms involved in cysteine elevations, including a selective dysregulation of enzymes responsible for its synthesis and degradation [48].

Another interesting observation in our systematic review and meta-analysis was the higher concentration of circulating ornithine in patients with stable COPD compared to healthy controls. As previously described (Figure 1), ornithine is the end product of the arginase 1 and 2 enzymes [60]. Therefore, an increase in ornithine concentrations is suggestive of an increased expression and/or activity of arginase which, in turn, reduces the availability of arginine as a NOS substrate for the synthesis of NO. However, this theory has been recently challenged by an elegant in vitro study investigating enzyme kinetics, which reported that the competition between arginase and NOS for the same substrate, arginine, does not occur in the presence of a maintained supply of extracellular arginine, which more adequately reflects the cellular physiological conditions. In the same study, the investigators reported that alterations in arginine transport and/or protein synthesis are more likely to influence NOS activity [141]. Pending additional studies investigating the possible involvement of arginase on NO synthesis, arginase upregulation has been reported in experimental models of COPD and clinical studies. For example, mice exposed to cigarette smoking for 13 weeks showed a significant increase in the expression of arginase [142]. Similar smoking-mediated increases in arginase expression have been observed in rabbits, with a concomitant reduction in NOS expression and activity [143]. Furthermore, treatment with arginase inhibitors significantly suppressed bronchial reactivity in patients with COPD [144]. An increased arginase activity has also been reported in platelets and erythrocytes in this group [145]. Pending confirmatory studies, this observation suggests that pharmacological strategies downregulating arginase might provide beneficial effects in COPD, independently of NO synthesis [146,147,148,149].

Our study had several strengths, including the comprehensive assessment of arginine, transsulfuration, and folic acid metabolomics in stable COPD and the robust evaluation of the risk of bias and the certainty of evidence for each studied metabolite. Limitations included the small group of selected studies for most metabolites, with the exception of homocysteine, which prevented sensitivity analysis, the assessment of publication bias, and the conduct of meta-regressions and subgroup analyses to investigate associations between the effect size and several clinical and demographic variables, e.g., age, sex, and markers of inflammation, and to identify possible sources of heterogeneity. Further studies are also necessary to investigate the potential pathophysiological role of citrulline and vitamin B_6_, given that our systematic search identified only one relevant study for each metabolite. Another significant limitation was the paucity of data reported in the selected studies regarding specific comorbidities, e.g., neurological and cardiovascular disease states, dietary patterns, and medications, factors which could also affect the concentrations of the studied metabolites [55,56,68,126,150,151,152,153,154,155,156]. At the same time, however, the assessment of the concentrations of folic acid, vitamin B_6_, and B_12_ may indirectly reflect dietary behaviours given their associations with specific food sources [157,158].

## 5. Conclusions

Our study showed significant alterations in the circulating concentrations of methionine, homocysteine, and cysteine (transsulfuration pathway), folic acid (folic acid pathway), and ADMA, SDMA, and ornithine (arginine pathway) in COPD. These alterations are suggestive of impaired NO synthesis and redox balance and may also explain the frequent occurrence of specific comorbidities, particularly atherosclerotic cardiovascular disease, in this patient group. Further research is warranted to confirm these findings, to investigate further associations between these metabolites and age, sex, markers of inflammation, specific comorbidities, dietary patterns, and medications, and to assess the effects of ADMA/homocysteine-lowering therapies and arginase inhibitors on lung function, symptom burden, disease progression, and mortality in COPD. 

## Figures and Tables

**Table 1:** Study characteristics.

	Healthy Controls					Patients with COPD				
Study	n	Age (Years)	M/F	Homocysteine Cysteine Methionine Vitamin B_6_ Vitamin B_12_ Folic Acid (Mean ± SD)	Arginine ADMA SDMA Ornithine Citrulline (Mean ± SD)	n	Age (Years)	M/F	Homocysteine Cysteine Methionine Vitamin B_6_ Vitamin B_12_ Folic acid (Mean ± SD)	Arginine ADMA SDMA Ornithine Citrulline (Mean ± SD)
Pow EM et al., 1998, The Netherlands [86]	8	64	NR	NR NR 28 ± 3 NR NR NR	90 ± 14 NR NR 61 ± 6 54 ± 7	12	66	NR	NR NR 26 ± 3.5 NR NR NR	90 ± 14 NR NR 74 ± 21 48 ± 6
Andersson A et al., 2001, Sweden [87]	29	64	14/15	14.1 ± 4.9 321 ± 50 NR NR NR NR	NR NR NR NR NR	19	68	8/11	17.9 ± 6.7 340 ± 52 NR NR NR NR	NR NR NR NR NR
Yoneda T et al., 2001, Japan [88]	30	NR	NR	NR NR 30.4 ± 7.3 NR NR	97.7 ± 22.8 NR NR 83.9 ± 19.7 NR	30	64	NR	NR NR 27.4 ± 5.4 NR NR	103 ± 21.6 NR NR 112.4 ± 32.1 NR
Kai S et al., 2006, Japan [89]	23	63	23/0	9.8 ± 3.0 NR NR NR NR NR	NR NR NR NR NR	24	71	24/0	12.6 ± 2.9 NR NR NR NR NR	NR NR NR NR NR
Seemungal TAR et al., 2007, England [90]	25	65	16/9	8.1 ± 2.2 NR NR NR NR NR	NR NR NR NR NR	29	69	23/6	10.7 ± 4.5 NR NR NR NR NR	NR NR NR NR NR
Abdallah GM et al., 2009, Egypt [91]	20	NR	12/8	7.6 ± 1.3 NR NR NR 335 ± 58 6.2 ± 3.0	NR NR NR NR NR	24	NR	18/6	9.4 ± 1.3 NR NR NR 299 ± 44 4.8 ± 2.9	NR NR NR NR NR
Fimognari FL et al., 2009, Italy [92]	29	71	21/8	11.9 ± 2.9 NR NR 9.1 ± 6.4 369 ± 211 3.0 ± 1.4	NR NR NR NR NR	42	71	36/6	14.8 ± 4.7 NR NR 5.6 ± 5.1 324 ± 144 2.3 ± 1.6	NR NR NR NR NR
Kahn NA et al., 2016, India [93]	30	52	13/17	15.2 ± 15.7 NR NR NR NR NR	NR NR NR NR NR	50	58	43/7	27.4 ± 27.9 NR NR NR NR NR	NR NR NR NR NR
Ruzsics I et al., 2016, Hungary [94]	30	51	15/15	NR NR NR NR NR NR	80 ± 3.5 0.35 ± 0.02 0.42 ± 0.02 NR NR	32	59	14/18	NR NR NR NR NR NR	106 ± 3.5 0.50 ± 0.03 0.65 ± 0.02 NR NR
Zinellu A et al., 2016, Italy [95]	43	73	34/9	NR NR NR NR NR NR	80 ± 16 0.50 ± 0.07 0.48 ± 0.18 NR NR	43	75	34/9	NR NR NR NR NR NR	64 ± 13 0.51 ± 0.13 0.51 ± 0.11 NR NR
Aydin M et al., 2017, Turkey [96]	30	64	21/9	NR NR NR NR NR NR	NR 47.9 ± 6.5 NR NR NR	58	62	48/10	NR NR NR NR NR NR	NR 70.7 ± 9.1 NR NR NR
Costanzo L et al., 2017, Italy [97]	35	74	16/9	NR NR NR NR NR NR	NR 3.18 ± 3.39 NR NR NR	41	74	23/18	NR NR NR NR NR NR	NR 3.19 ± 2.87 NR NR NR
Urban MH et al., 2017, Austria [98]	40	62	14/26	NR NR NR NR NR NR	NR 0.54 ± 0.10 NR NR NR	60	64	32/28	NR NR NR NR NR NR	NR 0.48 ± 0.09 NR NR NR
Lin CH et al., 2018, Taiwan [99]	36	71	36/0	14.6 ± 4.4 NR NR NR 628 ± 323 12.9 ± 6.3	NR NR NR NR NR	59	71	59/0	16.1 ± 18.4 NR NR NR 653 ± 327 11.0 ± 6.5	NR NR NR NR NR
Moayyedkazemi A et al., 2018, Iran [100]	51	66	29/22	18.2 ± 9.5 NR NR NR NR NR	NR NR NR NR NR	40	67	22/18	19.5 ± 9.5 NR NR NR NR NR	NR NR NR NR NR
Telo S et al., 2018, Turkey [101]	40	69	31/9	NR NR NR NR NR NR	NR 0.42 ± 0.04 NR NR NR	80	69	65/15	NR NR NR NR NR NR	NR 0.43 ± 0.05 NR NR NR
Csoma B et al., 2019, Hungary [102]	15	51	6/9	NR NR NR NR NR NR	NR NR 0.45 ± 0.14 NR NR	29	63	13/16	NR NR NR NR NR NR	NR NR 0.53 ± 0.14 NR NR
Kuo WK et al., 2018, Taiwan [103]	44	53	36/8	NR NR NR NR NR NR	NR NR NR 90.6 ± 18.3 NR	75	72	67/8	NR NR NR NR NR NR	NR NR NR 99.1 ± 32.3 NR
Wei B et al., 2020, China [104]	50	58	28/22	7.5 ± 2.7 NR NR NR NR NR	NR NR NR NR NR	150	62	90/60	11.7 ± 2.7 NR NR NR NR NR	NR NR NR NR NR
Yu T et al., 2020, China [105]	121	59	77/44	8.3 ± 4.4 NR NR NR NR NR	NR NR NR NR NR	119	59	86/33	14.8 ± 6.9 NR NR NR NR NR	NR NR NR NR NR
Zinellu A et al., 2020, Italy [106]	54	73	40/14	13.0 ± 3.7 317 ± 74 NR NR NR NR	NR NR NR NR NR	54	73	40/14	15.5 ± 3.8 340 ± 52 NR NR NR NR	NR NR NR NR NR

Legend: NR, not reported; M, male; F, female; COPD, chronic obstructive pulmonary disease; ADMA, asymmetric dimethylarginine; SDMA, symmetric dimethylarginine. The concentration of homocysteine, cysteine, methionine, arginine, citrulline, SDMA, and ornithine is expressed in µmol/L. The concentration of ADMA is expressed in µmol/L or ng/mL. The concentration of vitamin B_6_ and folic acid is expressed in ng/mL. The concentration of vitamin B_12_ is expressed in pg/mL.

## Data Availability

The relevant data are available from A.Z. upon reasonable request.

## Supplementary Materials

The following supporting information can be downloaded at: https://www.mdpi.com/article/10.3390/cells12172180/s1, Table S1: PRISMA 2020 for abstracts checklist; Table S2: PRISMA 2020 checklist; Table S3: The Joanna Briggs Institute critical appraisal checklist.
