# Supplementary material for: Arginine, Transsulfuration, and Folic Acid Pathway Metabolomics in Chronic Obstructive Pulmonary Disease: A Systematic Review and Meta-Analysis
Source: Cells. 2023 Aug 30;12(17):2180. doi: 10.3390/cells12172180 (PMC10486395; doi:10.3390/cells12172180)
Supplement: Supplementary file 1 [file cells-12-02180-s001.zip › Supplementary_Table_S3.pdf]

Supplementary Table S3. The Joanna Briggs Institute critical appraisal checklist.

Study	Were the criteria for inclusion clearly defined?	Were the subjects and the setting described in detail?	Was the exposure measured in a valid and reliable way?	Were objective, standard criteria used for measurement of the condition?	Were confounding factors identified?	Were strategies to deal with confounding factors stated?	Were the outcomes measured in a valid and reliable way?	Was appropriate statistical analysis used?	Risk of bias
Pow EM [1]	Yes	Yes	Yes	Yes	Yes	Yes	Yes	Yes	Low
Andersson A [2]	Yes	Yes	Yes	Yes	Yes	Yes	Yes	Yes	Low
Yoneda T [3]	Yes	Yes	Yes	Yes	No	No	Yes	Yes	Low
Kai S [4]	Yes	Yes	Yes	Yes	No	No	Yes	Yes	Low
Seemungal TAR [5]	Yes	Yes	Yes	Yes	Yes	Yes	Yes	Yes	Low
Abdallah GM [6]	Yes	Yes	Yes	Yes	No	No	Yes	Yes	Low
Fimognari FL [7]	Yes	Yes	Yes	Yes	Yes	Yes	Yes	Yes	Low
Kahn NA [8]	Yes	Yes	Yes	Yes	No	No	Yes	Yes	Low
Ruzsics I [9]	Yes	Yes	Yes	Yes	Yes	Yes	Yes	Yes	Low
Zinellu A [10]	Yes	Yes	Yes	Yes	Yes	Yes	Yes	Yes	Low
Aydin M [11]	Yes	Yes	Yes	Yes	Yes	Yes	Yes	Yes	Low
Costanzo L [12]	Yes	Yes	Yes	Yes	Yes	Yes	Yes	Yes	Low
Urban MH [13]	Yes	Yes	Yes	Yes	Yes	Yes	Yes	Yes	Low
Lin CH [14]	Yes	Yes	Yes	Yes	Yes	Yes	Yes	Yes	Low
Moayyedkazemi A [15]	Yes	Yes	Yes	Yes	No	No	Yes	Yes	Low
Telo S [16]	Yes	Yes	Yes	Yes	No	No	Yes	Yes	Low
Csoma B [17]	Yes	Yes	Yes	Yes	No	No	Yes	Yes	Low
Kuo WK [18]	Yes	Yes	Yes	Yes	Yes	Yes	Yes	Yes	Low
Wei B [19]	Yes	Yes	Yes	Yes	No	No	Yes	Yes	Low
Yu T [20]	Yes	Yes	Yes	Yes	No	No	Yes	Yes	Low
Zinellu [21]	Yes	Yes	Yes	Yes	Yes	Yes	Yes	Yes	Low

References

1. Pouw, E.M.; Schols, A.M.; Deutz, N.E.; Wouters, E.F. Plasma and muscle amino acid levels in relation to resting energy expenditure and inflammation in stable chronic obstructive pulmonary disease. *Am J Respir Crit Care Med* **1998**, *158*, 797-801, doi:10.1164/ajrccm.158.3.9708097.
2. Andersson, A.; Ankerst, J.; Lindgren, A.; Larsson, K.; Hultberg, B. Hyperhomocysteinemia and changed plasma thiol redox status in chronic obstructive pulmonary disease. *Clin Chem Lab Med* **2001**, *39*, 229-233, doi:10.1515/CCLM.2001.036.
3. Yoneda, T.; Yoshikawa, M.; Fu, A.; Tsukaguchi, K.; Okamoto, Y.; Takenaka, H. Plasma levels of amino acids and hypermetabolism in patients with chronic obstructive pulmonary disease. *Nutrition* **2001**, *17*, 95-99, doi:10.1016/s0899-9007(00)00509-8.
4. Kai, S.; Nomura, A.; Morishima, Y.; Ishii, Y.; Sakamoto, T.; Hegab, A.E.; Sekizawa, K. The effect of smoking-related hyperhomocysteinemia on spirometric declines in chronic obstructive pulmonary disease in elderly Japanese. *Arch Gerontol Geriatr* **2006**, *42*, 117-124, doi:10.1016/j.archger.2005.06.003.
5. Seemungal, T.A.; Lun, J.C.; Davis, G.; Neblett, C.; Chinyepi, N.; Dookhan, C.; Drakes, S.; Mandeville, E.; Nana, F.; Setlhake, S.; et al. Plasma homocysteine is elevated in COPD patients and is related to COPD severity. *Int J Chron Obstruct Pulmon Dis* **2007**, *2*, 313-321, doi:10.2147/copd.s2147.
6. Abdallah, G.M.; Abdullah, A.; Omran, G.A.; Mariee, A.D. Serum Homocystein Level In COPD Patients: Possible Beneficial Effect Of Antioxidants. *Res J Med Med Sci* **2009**, *4*, 306-310.
7. Fimognari, F.L.; Loffredo, L.; Di Simone, S.; Sampietro, F.; Pastorelli, R.; Monaldo, M.; Violi, F.; D'Angelo, A. Hyperhomocysteinaemia and poor vitamin B status in chronic obstructive pulmonary disease. *Nutr Metab Cardiovasc Dis* **2009**, *19*, 654-659, doi:10.1016/j.numecd.2008.12.006.
8. Khan, N.A.; Saini, H.; Mawari, G.; Kumar, S.; Hira, H.S.; Daga, M.K. The Effect of Folic Acid Supplementation on Hyperhomocysteinemia and Pulmonary Function Parameters in Chronic Obstructive Pulmonary Disease: A Pilot Study. *J Clin Diagn Res* **2016**, *10*, OC17-OC21, doi:10.7860/JCDR/2016/21322.8927.
9. Ruzsics, I.; Nagy, L.; Keki, S.; Sarosi, V.; Illes, B.; Illes, Z.; Horvath, I.; Bogar, L.; Molnar, T. L-Arginine Pathway in COPD Patients with Acute Exacerbation: A New Potential Biomarker. *COPD* **2016**, *13*, 139-145, doi:10.3109/15412555.2015.1045973.
10. Zinellu, A.; Fois, A.G.; Sotgia, S.; Sotgiu, E.; Zinellu, E.; Bifulco, F.; Mangoni, A.A.; Pirina, P.; Carru, C. Arginines Plasma Concentration and Oxidative Stress in Mild to Moderate COPD. *PLoS One* **2016**, *11*, e0160237, doi:10.1371/journal.pone.0160237.
11. Aydin, M.; Altintas, N.; Cem Mutlu, L.; Bilir, B.; Oran, M.; Tulubas, F.; Topcu, B.; Tayfur, I.; Kucukyalcin, V.; Kaplan, G.; et al. Asymmetric dimethylarginine contributes to airway nitric oxide deficiency in patients with COPD. *Clin Respir J* **2017**, *11*, 318-327, doi:10.1111/crj.12337.
12. Costanzo, L.; Pedone, C.; Battistoni, F.; Chiurco, D.; Santangelo, S.; Antonelli-Incalzi, R. Relationship between FEV(1) and arterial stiffness in elderly people with chronic obstructive pulmonary disease. *Aging Clin Exp Res* **2017**, *29*, 157-164, doi:10.1007/s40520-016-0560-3.
13. Urban, M.H.; Eickhoff, P.; Funk, G.C.; Burghuber, O.C.; Wolzt, M.; Valipour, A. Increased brachial intima-media thickness is associated with circulating levels of asymmetric dimethylarginine in patients with COPD. *Int J Chron Obstruct Pulmon Dis* **2017**, *12*, 169-176, doi:10.2147/COPD.S118596.
14. Lin, C.H.; Chen, K.H.; Chen, C.M.; Chang, C.H.; Huang, T.J.; Lin, C.H. Risk factors for osteoporosis in male patients with chronic obstructive pulmonary disease in Taiwan. *PeerJ* **2018**, *6*, e4232, doi:10.7717/peerj.4232.
15. Moayyedkazemi, A.; Rahimirad, M. Evaluating the Serum Homocysteine Level in the Patients with Chronic Obstructive Pulmonary Disease and its Correlation with Severity of the Disease. *J Res Med Dent Sci* **2018**, *6*.
16. Telo, S.; Kirkil, G.; Kuluozturk, M.; Balin, M.; Deveci, F. Can ADMA play a role in determining pulmonary hypertension related to chronic obstructive pulmonary disease? *Clin Respir J* **2018**, *12*, 1433-1438, doi:10.1111/crj.12675.
17. Csoma, B.; Bikov, A.; Nagy, L.; Toth, B.; Tabi, T.; Szucs, G.; Komlosi, Z.I.; Muller, V.; Losonczy, G.; Lazar, Z. Dysregulation of the endothelial nitric oxide pathway is associated with airway inflammation in COPD. *Respir Res* **2019**, *20*, 156, doi:10.1186/s12931-019-1133-8.
18. Kuo, W.K.; Liu, Y.C.; Chu, C.M.; Hua, C.C.; Huang, C.Y.; Liu, M.H.; Wang, C.H. Amino Acid-Based Metabolic Indexes Identify Patients With Chronic Obstructive Pulmonary Disease And Further Discriminates Patients In Advanced BODE Stages. *Int J Chron Obstruct Pulmon Dis* **2019**, *14*, 2257-2266, doi:10.2147/COPD.S220557.

19. Wei, B.; Tian, T.; Liu, Y.; Li, C. The diagnostic value of homocysteine for the occurrence and acute progression of chronic obstructive pulmonary disease. *BMC Pulm Med* **2020**, *20*, 237, doi:10.1186/s12890-020-01265-w.
20. Yu, T.; Niu, W.; Niu, H.; Duan, R.; Dong, F.; Yang, T. Chitinase 3-like 1 polymorphisms and risk of chronic obstructive pulmonary disease and asthma in a Chinese population. *J Gene Med* **2020**, *22*, e3208, doi:10.1002/jgm.3208.
21. Zinellu, A.; Zinellu, E.; Sotgiu, E.; Fois, A.G.; Paliogiannis, P.; Scano, V.; Piras, B.; Sotgia, S.; Mangoni, A.A.; Carru, C.; et al. Systemic transsulfuration pathway thiol concentrations in chronic obstructive pulmonary disease patients. *Eur J Clin Invest* **2020**, e13267, doi:10.1111/eci.13267.
